# Advanced-stage presentation of cancer at the time of diagnosis and its associated factors among adult cancer patients at Northwest Amhara comprehensive specialized hospitals, Northwest Ethiopia 2022

**DOI:** 10.1186/s12885-024-11835-4

**Published:** 2024-01-12

**Authors:** Likinaw Abebaw Wassie, Chilot Kassa Mekonnen, Yenework Mulu Tiruneh, Mamaru melkam, Eyayaw Ashete Belachew, Alebachew Ferede Zegeye

**Affiliations:** 1 Department of Medical Nursing, School of Nursing, College of Medicine and Health Sciences, University of Gondar, Gondar, Ethiopia; 2 Department of Surgical Nursing, School of Nursing, College of Medicine and Health Sciences, University of Gondar, Gondar, Ethiopia; 3 Department of Psychiatry, College of Medicine and Health Sciences, University of Gondar, Gondar, Ethiopia; 4 Department of clinical pharmacology of pharmacy, College of Medicine and Health Sciences, University of Gondar, Gondar, Ethiopia

**Keywords:** Cancer, Prevalence, Late-stage, Gondar, Ethiopia

## Abstract

**Introduction:**

Screening of cancer is the maximum effort and critical element for providing health related care in order to decrease cancer related dealt because of the disease burden is in its advanced stages. Unfortunately, advanced-stage presentation and late diagnosis of cancers endure a problem in low-income countries including Ethiopia. However, there is scarcity of published articles about the problem in Ethiopia.

**Objective:**

This study aimed to assess the prevalence of advanced-stage -stage presentation of cancer at the time of diagnosis and associated factors among adult cancer patients at Northwest Amhara comprehensive Specialized Hospitals, oncology treatment units, Northwest Ethiopia, 2022.

**Methods:**

An institution based cross-sectional study was conducted in Northwest Amhara public referral hospitals on 422 study participants. A systematic random sampling technique was performed. The data were collected through face to face interview and document review via structured, pretested questionnaires. Epi. Data version 4.6 and Stata version 14.0 software’s were used for data entry and analysis respectively. Logistic regression were carried out to recognize factors associated with advanced-stage -stage presentation of cancer at the time of diagnosis. Adjusted odds ratio with a 95% confidence interval were used to measure the strength of association. Variables having *p*-value less than 0.2 in bivariable analysis were entered in to multivariable analysis; variables with a *p*-value < 0.05 were declared significantly associated with advanced-stage -stage presentation of cancer at the time of diagnosis.

**Results:**

The overall prevalence of advanced-stage presentation of cancer at the time of diagnosis was found to be 67.57%. Age ≥ 60 years old patients (AOR = 6.10, 95%: (1.16–32.1)), patients had have a feeling of burden (AOR = 1.82, 95%, CI: (1.04–3.20)), and cancer patients with comorbidity illness (AOR = 2.40, 95%, CI: (1.40–4.12)) were significantly associated with advanced-stage presentation of cancer at the time of diagnosis.

**Conclusion:**

The prevalence of advanced-stage presentation of cancer at the time of diagnosis was found to be high. Its better, health care providers in oncology treatment facilities need to give special attention to older patients, having feeling of burden and cancer patient with comorbidity to reduce the risk of developing late stage presentation of cancer.

## Introduction

Cancer is one of the foremost fatal diseases of recent times that causes numerous deaths annually [1]. It has been estimated that more than 19.3 million new cancer cases were detected and recognized a moment ago, a leading cause to around 10 million deaths in 2020 [2]. Sub-Saharan African (SSA) countries are burdened with the incidence of one or more types of cancer; commonly they are on the list of developing countries [3]. Practically 70% of all cancer related mortalities have been in low- and middle-income countries, comprising Ethiopia [4, 5].

Patient delay is defined as the time between recognizing a cancer symptom and the first visit to a healthcare professional [6]. It accounts for the maximum proportion of delay time in the alleyway from clinical futures detection to the initiation of cancer management [7]. Advance-stage presentation at the time of diagnosis is a major factor contributing to excess deaths in England, laterally with the rest of the United Kingdom, from breast [8], bowel, and lung cancer [9].

A population-based study conducted in different European countries reported that the prevalence of advanced-stage of lung cancer at the time of diagnosis was approximately ranged from 46.8 to 82% [10]. A Previous systematic review revealed that the prevalence rate for breast cancer patients in Sub-Saharan Africa (SSA) is detected with advanced stage of disease ranging from 30.3 to 100% and prime cause of low continuity [11]. Around 64% of patients with cervical cancer were detected with delayed-stage of the disease (III and IV). Approximately 45% of cervical cancer patients existed with an advanced stage (Stage III) and utmost (91.4%) histology nature was squamous cell carcinoma [12].

Numerous studies have indicated that the advanced-stage presentation of cancer at the time of diagnosis is a major factor affecting the longstanding health and well-being of cancer patients [13–15]. Late manifestation with symptoms among patients with cancer is more likely to be a cause of advanced stage at the time of diagnosis and, in so doing, relatively worsen survival rate [16].

Globally, cancer control strategies increasingly encompass the early diagnosis of symptomatic cancer alongside primary prevention policies and screening programs [17]. Revealing at an early stage rises the likelihood that cancers can be preserved successfully, decreasing the risk of morbidity and mortality and improving long-term prediction [18].

Several studies have reported that factors contributing to the advanced stage of presentation are sociodemographic status, low socioeconomic status, co-morbidity, type of cancer [19], tumor biology [20], stage of disease at the time of diagnosis [21], beliefs about cancer [22], access to therapy, and type of treatment were more likely to be associated with the late-stage presentation of cancer [23].

Early diagnosis of cancer is the most effective mechanism for timely handling and reducing mortality rate even though the fact that disease prevalence is in its earliest stages. Regrettably, late presentation and delayed diagnosis of cancers leftovers are a problem in low and middle-income countries. Regardless of this, as per the investigators’ knowledge, there is little data known about the problem in Northwest Amhara as well as in Ethiopia. Therefore, this study aimed to determine the prevalence of advanced-stage presentation and associated factors among adult patients with cancer.

## Methods

### Study design and period

A facility-based cross-sectional study was employed among adult cancer patients from March 15 to May 15, 2022.

### Study area

The study was accompanied in Northwest Amhara Comprehensive Specialized Hospitals. There are a total of eight Comprehensive Specialized Hospitals in the Amhara Region of which five; are Debre Markos, Felege Hiwot, Tibebe Gion, Debre Tabor, and the University of Gondar originate in the Northwest of Amhara. Each Comprehensive Specialized Hospital serves 3.5–5 million people [24]. Of the five Referral Hospitals, the two (University of Gondar Comprehensive Specialized Referral Hospital (UoGCSH) and Felege Hiwot Comprehensive Specialized Hospital (FHRH)) have oncology treatment units. Those two Referral Hospitals are placed in the Amhara Regional State, Northwest Ethiopia 738 km and 565 km away from the capital city of Ethiopia: Addis Ababa respectively. Those oncology treatment units provide chemotherapy, hormonal therapy and surgical treatment for patients with cancer. There are a total of 980 cancer patients in the two Referral Hospitals. The oncology treatment unit of UoGCSH was established in 2014 G.C and at this time has 500 cancer patients and 17 beds for the management of cancer patients. Whereas the oncology treatment unit of FHCSH was established in 2016 G.C and has 480 cancer patients; presently has 18 beds for inpatient treatment of cancer patients. A one-month average number of cancer patients who had follow-up treatments in UoGCSH and FHCSH were 250 and 240 respectively.

### Study participants

The study population were all adult cancer patients who were 18 years old and above and who had follow up at Northwest Amhara Comprehensive Specialized Hospitals were included in the study. Those adult cancer patients with incomplete medical records were excluded from the study.

### Sample size and Sampling Procedure

The sample size was determined by using the single population proportion formula; Bearing in mind the following assumption: 95% confidence interval (CI), 50% proportion of late-stage presentation of cancer at the time of diagnosis, have no previous study conducted, and 5% margin of error. The final sample size was 422 by considering a 10% non-respondent rate. A systematic random sampling method was in employment to select study participants in the study area by using the order of the case coming. The sample size was proportionally allocated for each selected Hospital based on the number of cancer patients. Based on the report obtained from the oncology treatment units; considering this, on average the total number of cancer patient who had follow up in two month is around 980. The first participant to be included in the study was selected by the lottery method, (K = N/n, where; K = the interval, N = total population, and n = sample size) and then, every 3 participants were interviewed.

### Operational definitions

**Late stage presentation-** participants who have presented or were diagnosed in late or advanced stages (stage III to IV) of cancer at first diagnosis [25, 26].

### Data collection instruments and procedures

The data were collected by using interviewer-administered and chart review through structured pretested questionnaires that are adapted from a questionnaire developed by a previous study [27–31], which contains four sections the first section contains eight questions regarding socio-demographic characteristics of the study participants, the second section contains seven questions related to clinical characteristics of a patient, the third questions regarding the nutritional status and the fourth sections related to behavioral factors of a patient with cancer.

### Data processing and analysis

After data collection the questionnaire was checked, coded, and using Epi data version 4.6, after being coded and analyzed using Stata version 14.0. Descriptive statistics such as percentage, frequency, mean and standard deviation were used. To display the findings; tables and bar graphs were used. All continuous variables were tested for normality using the Shapiro-Wilk test and its distribution was also checked. The model fitness was also checked by Hosmer-Lemeshow goodness of fit with the *P*-value of > 0.05. The binary logistic regression analysis model was used. In the Bivariable analysis those variables having a *p*-value of < 0.25 were considered to be computed into the multivariable analysis to minimize the possible confounding factors for late-stage presentation of cancer. Variables having *p*-values less than 0.05 with 95% CI considered as statically significant with late-stage presentation of cancer at the time of diagnosis. Multicollinearity and model goodness of fit test were checked by using variance inflation factors (VIF = 1.15–4.80) and Hosmer and Lemeshow goodness of fit test (*p* = 0.13) respectively.

### Data quality control

Pretest was conducted among 5% [21] patients outside of the study area using structured questionnaire prior to the data collection time. Moreover, the data collectors and supervisors were trained for one day before data collection about the ultimate objectives and concepts of the questionnaire, the required ethical conduct, the secrecy of the information, the rights of the participants and the technique of choosing the study participants from each oncology treatment unit. Daily communication and on the spot correction of the data was done between principal investigator, supervisors and the data collectors throughout the data collection process. Initially the tool was translated into local language (Amharic) earlier data collection. Then back translation into English language was done to see its consistency. Uniformity was crisscrossed by a back-translation by another expert fluent both in English and in local languages. At the end of each data collection day, the supervisors were checked for completeness or fulfillment of the questionnaires and the quality of the recorded information.

### Ethical consideration

Ethical clearance was obtained from the School of Nursing research ethical review commute on the behalf of the University of Gondar institutional review board. Written permission letters were obtained from Hospital managers. Participants were informed about the purpose of the study and written informed consent was obtained from them. Confidentiality was maintained by omitting direct personal identifiers on the questionnaire, using code numbers, storing data locked with a password, and not misusing or disclosing their information. Participants were also be informed that participation was voluntary and they have the right to withdraw from the study participation at any stage if they are not comfortable with the investigation. The issue of privacy and confidentiality was strictly maintained.

## Results

### Socio-demographic characteristics

A total of 422 study participants were enrolled in the study with a response rate of 407 (96.45%). The Median age of the study participant was 35 with an Inter-Quartile Range (IQR) of 28-46.5 years. Nearly one-third 128 (31.45%) of them were between the age of 30–39 years. More than half 259 (63.64%) of the study participants were male and the majority 327 (79.12%) of the respondents were followers of Orthodox Christianity. Most 395 (95.87%) of the respondents were Amhara by ethnicity. A little bit less than three fourth 288 (70.76%) of the study participants were married on whose marital status and more than half 232 (56.31%) study participants were urban dwellers (Table 1).

**Table 1:** Socio-demographic characteristics of adult patients with cancer in Northwest Amhara Comprehensive Specialized Hospitals, oncology treatment units, 2021. (*n* = 407)

Variables	Category	Frequency(n)	Percent (%)
Age	< 30	114	28.01
	30–39	128	31.45
	40–49	80	19.66
	50–59	62	15.23
	>=60	23	5.65
Sex	Male	259	63.64
	Female	148	36.36
Marital status	Single	107	26.29
	Married	288	70.76
	Others	12	2.95
Religion	Orthodox	322	79.12
	Muslim	80	19.66
	Others	5	1.23
Ethnicity	Amhara	390	95.82
	Others	17	4.18
Residence	Urban	245	60.20
	Rural	162	39.80
Educational status	No formal education	101	24.82
	Primary school	76	18.67
	Secondary school	141	34.64
	Collage and above	89	21.87
Occupation	Governmental Employed	58	14.25
	NGO employed	99	24.32
	Farmer	106	26.04
	Merchant	41	10.07
	Student	52	12.78
	Housewife	43	10.57
	Others	8	1.97
Average monthly income in ETB	< 1000	132	32.43
	1001–2000	61	14.99
	2001–3000	49	12.04
	>=3001	165	40.54

### Clinical-related characteristics of patients

Among a total of 407 cancer cases, around 31 types of cancer have gotten; breast cancer 88 (21.62%), cervical cancer 53 (13.02%), gastric cancer 30 (7.37%), and else. Closely two-thirds of 275 (67.57%) the study participants were have had advanced clinical stages of cancer (stage III& IV). Most 410 (99.51%) of the respondents were taking cancer treatment and the majority 363 (89.41%) of patients were taking chemotherapy. Nearly one-third of 120 (29.48%) of the patient have had known comorbid illness. More than one-third of 39 (32.50%) patients had hypertension. A little bit less than the majority 319 (78.38%) of the respondents were feeling burdened. More than three fourth 308 (75.68%) and almost all 399 (98.03%) of the study participants were not alcohol users and cigarette smokers respectively. (Table 2)

**Table 2:** Clinical-related characteristics of adult cancer patients in Northwest Amhara Comprehensive Specialized Hospitals, oncology treatment units, 2021. (*n* = 407)

Variables	Category	Frequency(n)	Percent (%)
Cancer type	Breast cancer	88	21.62
	Cervical cancer	53	13.02
	Gastric cancer	30	7.37
	Colonic cancer	30	7.37
	Others	206	50.62
Clinical stage	Early	132	32.43
	Advanced	275	67.57
Duration since diagnosis in months	<=12	258	63.39
	< 12	149	36.61
Does the patient take treatment?	Yes	405	99.51
	No	2	0.49
Duration since the start of treatment in months	<=6	293	72.35
	> 6	112	27.65
Type of treatment	Chemotherapy	363	89.41
	Hormonal therapy	25	6.16
	Mixed	13	3.20
	Surgery only	5	1.23
Cycle of chemotherapy	Two	51	16.45
	Three	52	16.77
	Four	70	22.58
	Five	82	26.45
	Six and above	55	17.74
ECOG performance	Grade 0	28	6.88
	Grade 1	78	19.16
	Grade 2	175	43.00
	Grade 3 & 4	126	30.96
Comorbidity	Yes	120	29.48
	No	287	70.52
Type of comorbidity	Anemia	29	24.17
	HIV/AIDS	18	15.00
	Hypertension	39	32.50
	Diabetes mellitus	22	18.33
	Others	12	10.00
Alcohol use	Yes	99	24.32
	No	308	75.68
Cigarette smoking	Yes	8	1.97
	No	399	98.03
Burden of patients	Yes	319	78.38
	No	88	21.62

### Factors associated with advanced-stage presentation

Bivariate analysis was conducted to identify factors associated with the advanced-stage presentation of cancer at the time of diagnosis among adult cancer patients. Age, marital status, education, occupation, residency, comorbidity, and burden of patients were significantly associated with the advanced-stage presentation of cancer at the time of diagnosis. Finally, multivariable analyses were carried out and age, comorbidity of disease, and burden of patients were significantly associated with advanced-stage presentation of cancer at the time of diagnosis among adult cancer patients. Age ≥ 60 years old patients were nearly six times more likely to develop advanced stage of cancer as compared to their counterparts (AOR = 6.10, 95%: (1.16–32.1)), patients had have a feeling of burden were nearly two times more likely to develop advanced stage of cancer as compared to had have no feeling burden (AOR = 1.82, 95%, CI: (1.04–3.20)), and cancer patients with comorbidity illness were nearly two times more likely to develop advanced stage of cancer as compared to had no comorbid illness (AOR = 2.40, 95%, CI: (1.40–4.12)). (Table 3)

**Table 3:** Factors associated with the advanced-stage presentation of adult cancer patients, July/2021. (*n* = 407)

Variables	Category	Advanced stage		COR (95% CI)	AOR(95%CI)	*P*-value
		Yes	No			
Age	<30	83	31	1	1	
	30-39	76	52	0.55(0.32-0.94)	0.67(0.33-1.36)	0.271
	40-49	54	26	0.78(0.42-1.45)	0.80(0.34-1.90)	0.619
	50-59	41	21	0.73(0.37-1.42)	0.78(0.31-1.92)	0.583
	>=60	16	7	3.92(0.87-17.7)	6.10(1.16-32.1)	0.033*
Marital status	Single	77	30	1	1	
	Married	187	101	0.72(0.44-1.17)	0.45(0.21-0.95)	0.055
	Others	7	5	4.29(0.53-34.7)	2.77(0.31-24.84)	0.362
Residency	Urban	152	93	1	1	
	Rural	123	39	1.93(1.24-3.01)	1.06(0.45-2.50)	0.890
Education	No formal education	78	23	1.72(0.91-3.27)	1.26(0.40-4.01)	0.698
	Primary(1-8)	57	19	1.53(0.77-3.01)	1.20(0.45-3.22)	0.716
	Secondary (9-12)	81	60	0.69(0.41-1.91)	0.40(0.18-0.90)	0.225
	Collage and above	59	30	1	1	
Occupation	Governmental employed	40	18	1	1	
	NGO employed	53	46	0.52(0.26-1.03)	0.67(0.28-1.64)	0.384
	Farmer	83	23	1.62(0.79-3.35)	1.22(0.32-4.63)	0.774
	Merchant	27	14	0.87(0.37-2.03)	1.36(0.46-4.02)	0.578
	Student	39	13	1.35(0.58-3.12)	1.30(0.35-4.85)	0.693
	Housewife	29	14	0.93(0.40-2.17)	1.01(0.29-3.44)	1.00
	Others	4	4	0.45(0.10-2.00)	0.19(0.03-1.05)	0.057
Burden of patients	Burdened	224	95	1.71(1.05-2.78)	1.82(1.04-3.20)	0.036*
	Not burdened	51	37	1	1	1
Comorbidity	Yes	56	59	1.75(1.08-2.85)	2.40(1.40-4.12)	0.001*
	No	170	127	1	1	

## Discussion

This study revealed the prevalence of advanced-stage presentation of cancer at the time of diagnosis and its associated factors among adult cancer patients who had follow up at Northwest Amhara Comprehensive Specialized Hospitals, oncology treatment units. The overall prevalence of advanced-stage presentation of cancer at the time of diagnosis was found to be 67.57% with (95% CI: 62.84–71.95). With regard to the overall prevalence of advanced-stage presentation of cancer at the time of diagnosis to get existing literature are challenging because published studies were cancer-site specific, However the prevalence of advanced-stage presentation of cancer at the time of diagnosis found in this study was higher than the results of a studies conducted in Washington 45.5%, Brazil (53.5%) among breast cancer patients [32], breast (28.94%), cervix (23.66%), and ovary (16.11%) in India, 46.0% in the Netherlands, possible justification for this difference is, it might be due to lack of awareness of patients about the symptoms of cancer in these country [7]. low awareness is often a reason which contributes towards low screening, thereby reducing the likelihood of early detection of cancer. This variation could also be due to differences in the socio-economic, cultural, and lifestyle differences between those countries. The possible reason may be due to health care delivery policy differences and the country’s priority to cancer treatment and prevention in the case of Washington, Netherlands and Brazil [33].

On the contrary, the prevalence of advanced-stage presentation of cancer at the time of diagnosis found in this study was lower than the finding of a study conducted in Uganda (80%) [34]. The possible reason for this discrepancy could be, due to study population difference which means Uganda study’s conducted on HIV infected patients. HIV epidemic has contributed to the increasing incidence of cancer in sub-Saharan Africa, where most patients with cancer present at an advanced stage [35]. Mover over the evidence suggests that the risk of cancer is 2–3,000 times higher among people with HIV than those who are not infected and up to 9% of people living with long-term HIV infection will develop advance stage of cancer over the course of their HIV care [36].

Regarding the associated factors, advanced-stage presentation of cancer at the time of diagnosis among cancer patients, patient’s age > = 60 years old were nearly six times more likely to present with advance stage of cancer as compared to their counterparts. This finding was supported by studies conducted in Estonia [37], Brazil [32], South India [23]. This might be due to some of the same biologic mechanisms that regulate aging also may be involved in the pathogenesis of age-related diseases such as cancer. Cancer development is a complex process that occurs over a span of many years. A life course approach is particularly well suited to understanding the contributions of various cancer risk factors over a person’s life span [38]. Cancer is a disease associated with aging the majority of cancer diagnoses and deaths occur in people older than 65 years. Furthermore, older adult patients have limitation to protect themselves from different risk factors of cancer due to aging process is associated with a decrease in physiologic reserve.

According to this finding, patients had have a feeling of burden were nearly two times more likely to develop advanced stage of cancer as compared to had have no feeling of burden. This might be due to the physical and psychological stress; so factors that affecting the physical and psychosocial well-being of cancer patient’s increase the severity, stage and presentation of cancer disease [39]. It also might be emotional distress a leading cause a patient having poor performance status towards different disease [40].

According to this study was found to be that, cancer patients with comorbidity illness were nearly two times more likely to develop advanced stage of cancer as compared to had no comorbid illness. Comorbidity potentially affects the development, stage at diagnosis, treatment, and outcomes of people with cancer. First, cancer and comorbid conditions share many common risk factors. Older age is associated with an increasing risk of late stage of cancer and of almost all other chronic conditions. Smoking, poor diet, lack of physical activity, obesity, and alcohol abuse are all risk factors for a range of common conditions as well as for advance stage of cancers [41]. Second, the biological mechanisms associated with comorbidity may predispose to late stage of cancer [42].

### Clinical implications

This paper have a lots of clinical contributions for different responsible bodies which means it is important for cancer patients to identify cancer symptom in order to get early treatment. It also used for policy makers to draft different strategies and policy to timely screening and preventions. Finally it has great implication for our country to reduce different cost and budget that break down for treatment and others necessary infrastructures to recover patient’s health.

### Limitation

The current study focused on overall cancer types, it make challenging to discuss with previous cancer specific site published studies. Most variables specifically behavioral related variables were self-reported and therefore may be affected by social desirability bias or defensive reactions.

## Conclusion

The prevalence of advanced-stage presentation of cancer at the time of diagnosis was found to be high. Age ≥ 60 years old patients, patients had have a feeling of burden, and cancer patients with comorbidity illness were factors associated with advanced-stage presentation of cancer at the time of diagnosis. Its better, health care providers in oncology treatment facilities need to recognize and screen, give special attention to older patients, having felling of burden of care and cancer patient with comorbidity to reduce the risk of developing late stage presentation of cancer.

## Data Availability

All relevant data are available within the manuscript.
